# Purification and Identification of Cholesterol Micelle Formation Inhibitory Peptides of Hydrolysate from High Hydrostatic Pressure-Assisted Protease Hydrolysis of Fermented Seabass Byproduct

**DOI:** 10.3390/ijms22105295

**Published:** 2021-05-18

**Authors:** Guan-Wen Chen, Hong-Ting Victor Lin, Li-Wen Huang, Chia-Hua Lin, Yu-Hsin Lin

**Affiliations:** 1 Department of Food Science, National Taiwan Ocean University, No. 2 Pei-Ning Road, Keelung 202, Taiwan; chengw@ntou.edu.tw (G.-W.C.); HL358@ntou.edu.tw (H.-T.V.L.); amykarenster@gmail.com (L.-W.H.); 2 Department of Biotechnology, National Formosa University, No. 64, Wunhua Rd, Yunlin 632, Taiwan; vicchlin@nfu.edu.tw; 3 Department of Food Science and Technology, Taipei University of Marine Technology, No. 212, Section 9, Yan Ping North Road, Taipei 111, Taiwan

**Keywords:** bile acid binding capacity, cholesterol micellar solubility, seabass byproduct, high hydrostatic pressure assisted protease hydrolysis, hypocholesterolemic peptide

## Abstract

This research focuses on the proteolytic capacity of sea bass byproduct (SB) and their hypocholesterolemic activity via the cholesterol micelle formation (CMF) inhibition. SB was fermented with seven mixed lactic acid bacteria for 5 h at 42 °C. The lactic fermented SB was hydrolyzed with Protease N for 6 h under HHP to obtain the SB hydrolysates (HHP-assisted Protease N hydrolysis after fermentation, F-HHP-PN6). The supernatant was separated from the SB hydrolysate and freeze-dried. As the hydrolysis time extended to 6 h, soluble protein content increased from 187.1 to 565.8 mg/g, and peptide content increased from 112.8 to 421.9 mg/g, while inhibition of CMF increased from 75.0% to 88.4%. Decreasing the CMF inhibitory activity from 88.4% to 42.1% by simulated gastrointestinal digestion (FHHP-PN6 was further hydrolyzed by gastrointestinal enzymes, F-HHP-PN6-PP) reduced the CMF inhibitory activity of F-HHP-PN6. Using gel filtration chromatography, the F-HHP-PN6-PP was fractioned into six fractions. The molecular weight of the fifth fraction from F-HHP-PN6-PP was between 340 and 290 Da, and the highest inhibitory efficiency ratio (IER) on CMF was 238.9%/mg/mL. Further purification and identification of new peptides with CMF inhibitory activity presented the peptide sequences in Ser-Ala-Gln, Pro-Trp, and Val-Gly-Gly-Thr; the IERs were 361.7, 3230.0, and 302.9%/mg/mL, respectively.

## 1. Introduction

A high cholesterol concentration can easily lead to hypercholesterolemia, a risk factor for coronary occlusion [1,2]. It is also associated with various cardiovascular diseases [3]. To prevent and slow hypercholesterolemia, cholesterol content should be reduced. Epidemiological studies have indicated that for every 1 mg/dL increase in total cholesterol concentration in the blood, the incidence of coronary artery disease increases by 2% [4]. One reason for an increase in plasma cholesterol concentration levels is the imbalance of endogenous cholesterol, dietary cholesterol, and excretion of bile acid and cholesterol in feces [5]. The cholesterol ingested with food combines with bile acid to form micelles during gastrointestinal absorption, thereby increasing its solubility and absorption in the intestines. Therefore, by inhibiting the solubility of diet-derived cholesterol in micelles, the emulsification of bile acid and cholesterol can be prevented; thus, cholesterol micelle formation (CMF) can be inhibited to lower cholesterol [6,7]. Bile acid sequestrants are widely used in lowering cholesterol, including cholestyramine, which reduces the reabsorption of endogenous cholesterol and bile acid and has been proved to be effective in reducing total cholesterol and low-density lipoprotein (LDL) as well as in reducing the mortality and incidence of cardiovascular diseases. However, cholestyramine produces side effects that can cause gastrointestinal discomfort, such as abdominal pain, heartburn, bloating, and constipation [8]. Therefore, searching for natural active substances such as peptides to replace drugs in disease management is essential.

In recent years, numerous studies have investigated protein hydrolysates or peptides as natural compounds for novel hypocholesterolemic activities. To obtain hypocholesterolemic peptides, many studies have focused on the protein hydrolysates of various food proteins such as β-lactoglobulin [6], beans [9,10,11], Atlantic horse mackerel [12], and clams [13,14,15]. Nagaoka et al. [6] demonstrated that the trypsin hydrolysate of β-lactoglobulin can inhibit CMF and purified and identified the first hypocholesterolemic peptide, Ile-Ala-Glu-Lys. In addition, a hydrolysate of chickpea protein hydrolyzed with Alcalase and Flavourzyme can inhibit CMF by up to 50% [9]. Moreover, after soy protein was hydrolyzed with Alcalase, the highest CMF inhibitory percentage was 48.6% (when the protein hydrolysis rate reached 18%). A mouse experiment also confirmed that this hydrolysate could reduce LDL in serum and cholesterol [10]. The hydrolysate was further purified, and its active peptide sequence was identified as Try–Gly–Ala–Pro–Ser–Ile [11]. Kwon et al. [16] also separated a tetrapeptide that lowered cholesterol from soy glycinin hydrolysate (Leu-Pro-Tyr-Pro). Our previous study determined that the protein hydrolysate obtained through the Protamax hydrolysis of freshwater clam (*Corbicula fluminea*) residual meat byproducts by using hot water extraction had a binding capacity of 35.9% with cholic acid and a CMF inhibitory capacity of 18.5% [13]. In addition, the protein hydrolysate of clam meat (16.6% in the diet) was fed to Sprague–Dawley rats with hyperlipidemia induced by high cholesterol for 4 weeks, and the results signified that the hydrolysate accompanied a significant reduction in cholesterol (cholesterol content in plasma and the liver were reduced by 26.1% and 50.5%, respectively) and blood lipids [14]. After further purification of the clam meat hydrolysate through column chromatography and identification of protein sequence, new peptide sequences that inhibited CMF were obtained, namely Val-Lys-Pro and Val-Lys-Lys [15]. Nonetheless, information on the hypocholesterolemic effects of protein hydrolysates and peptides is still scarce. Therefore, further studies on the hypocholesterolemic activity of protein hydrolysates, novel peptides, and identification of their peptide sequences are necessary to understand their possible mechanism of action.

Using protease hydrolysis to produce active peptides is a common preparation method. In addition, many novel processing methods are available to assist or combine enzyme hydrolysis for improving the yield and biological activity of active peptides, such as those involving lactic acid bacteria, ultrasound, and high hydrostatic pressure (HHP). Lactic acid bacteria can not only reduce the undesirable bitter peptides produced in protease hydrolysis but also enhance the biological activity of peptides [17,18,19]. An HHP-assisted enzyme extraction technology (50–200 MPa) can improve the extraction rate of biologically active compounds in food [20]. A possible reason is that pressure can change the protein structure, causing it to unfold and expose the compressed solution and the cleavage sites that the enzyme can bind to, thereby increasing the solute concentration and the collision rate between the enzyme and the protein [21,22], thereby inducing enzyme activity to increase the protein hydrolysis rate and the release of active peptides [23]. Current study of the use of HHP to assist enzyme hydrolysis to produce active peptides is limited but includes β-lactoglobulin [24], ovalbumin [25], chickpea protein [26], and pinto bean protein [27]. The extraction of HHP-facilitated protease hydrolysis has also been used to hydrolyze softshell turtle meat to increase the contents of soluble solids and peptides in protein hydrolysates [20,28]. Applying this technology in the extraction of edible bird’s nest can also increase the concentration and yield of sialic acid [29].

Sea bass (*Lates calcarifer*) is a common, affordable fish in the Asian market. In 2016 and 2017, the total production volume of Taiwanese sea bass was 39,358 metric tons, and the total export volume (including entire sea bass and sea bass fillets) was approximately 14,334 metric tons, with an output value of US$88.8 million. The export volume of sea bass fillets was 9,321 metric tons, accounting for approximately 65% of the total export volume, and its output value was approximately US$72 million [30]. However, the fish product industry often uses only the fish fillets when processing fish, which results in the production of byproducts including fish heads, bones, scales, tails, and internal organs. These fish byproducts cause environmental problems if they are not properly treated. In addition, these byproducts are a good source of protein and polyunsaturated fat [31,32]. However, no studies have conducted a correlation analysis of the hypocholesterolemic effect of the bioactive peptides of sea bass extract. If sea bass byproducts with high protein content can be used as raw material to develop dietary supplements, their use and commercial value are expected to increase.

Therefore, we selected sea bass byproducts as raw material. The byproducts were first fermented with lactic acid bacteria, and then HHP was used to facilitate commercial protease hydrolysis to enhance the reaction between proteases and substrates and to increase the active peptide content in sea bass hydrolysates. The hydrolysates were then subjected to a simulated in vitro gastrointestinal digestion test to evaluate their CMF inhibitory capacity and bile acid-binding capacity. The biologically active peptides were purified through size exclusion chromatography and reversed-phase high-performance liquid chromatography (RP-HPLC), and the amino acid sequences of peptides were identified to explore the correlation between active peptides in hydrolysates and hypocholesterolemic activity.

## 2. Results and Discussion

### 2.1. Effects of HHP and Enzyme Type on Chemical Composition of Hydrolysates and CMF-Inhibitory Capacity

To select suitable proteases for releasing peptides with high CMF-inhibitory activity from sea bass byproducts, we used nine commercial proteases (i.e., Alcalase, Flavourzyme, Peptidase R, Protamex, Protease M, Protease N, Protin NY100, Protin SD-AY10, and Umamizyme G) for protease hydrolysis under HHP of 100 MPa at 50 °C for 24 h and evaluated the inhibitory activity of the hydrolysate against CMF. The CMF inhibitor was the positive control group of the cholestyramine, with an inhibitory percentage of 98.0%. The CMF inhibitory percentage of each sea bass byproduct hydrolysate hydrolyzed through HHP-assisted protease hydrolysis was 19.4%, 0.8%, 21.4%, 16.5%, 22.8%, 30.1%, 19.5%, 18.5%, and 20.8%, respectively. Among them, the Protease N, Protease M, and Peptidase R groups had a higher CMF inhibitory capacity (data not shown). Therefore, these three groups of enzymes were used for further experimental analysis.

Sea bass byproducts were hydrolyzed by Protease N, Protease M, or Peptidase R under atmospheric pressure (0.1 MPa) and HHP (100 MPa) at 50 °C for 24 h to analyze the effect of hydrolysates with different enzymes under different pressures on soluble protein, peptide, and free amino acid content. The results revealed that soluble protein content was higher in the Protease N group—particularly, highest in the HHP group, with a value of 696.8 mg/g (Table 1), which was increased by 42.4 mg/g (1.1 times) from that under atmospheric pressure. A related study noted that the reaction of enzymes to substrate was specific. Protease N is an endonuclease that acts on proteins with larger molecular weights and increases soluble protein content [33]. Therefore, the Protease N group exhibited higher soluble protein content. In addition, the HHP-assisted protease hydrolysis changed the protein structure and caused denaturation, which in turn exposed new cleavage sites to enhance the effect of protein hydrolysis, increasing the reaction rate between the substrate protein and the enzyme [22,34]. Therefore, compared with the atmospheric pressure group, the HHP-assisted Protease N hydrolysis may enhance the hydrolysis of the protease and sea bass protein, giving the soluble protein content an increasing trend.

In the peptide content trends, we observed in the HHP group that the peptide content of Protease M and that of Peptidase R (908.6 and 806.4 mg/g) were significantly higher than that of the atmospheric pressure group (865.0 and 737.0 mg/g), which represented increases by approximately 1.1 to 1.2 times, and Protease M in HHP group exhibited the highest peptide content (Table 1). However, the free amino acid content also demonstrated the same trend (Table 1). Lahl and Brum [33] demonstrated that Protease M and Peptidase R are exonucleases, which can hydrolyze proteins with smaller molecular weights and thereby increase the content of peptides and of free amino acids. Bamdad et al. [35] discerned that the hydrolysate of β-lactoglobulin prepared by an HHP-assisted protease, Savinase, at 100 MPa had a molecular weight range of 500–1500 Da and a peptide content of <500 Da of 63.44% and 0.26%, respectively, which increased the peptide percentage of small molecular weights for approximately 1.8 and 1.6 times higher than the atmospheric pressure group (0.1 MPa, peptide content of 34.84% and 0.16%). The possible reason was that the process of protease hydrolysis facilitated by HHP was conducive to increasing the content of small molecular weight peptides [35].

This study explored the effect of hydrolysates of three proteases hydrolyzed under HHP and atmospheric pressure for 24 h on CMF-inhibitory capacity. Cholestyramine acted as the positive control, with an inhibitory capacity of 98.6 ± 0.6%. According to Table 1, the three protease groups under HHP were more capable of inhibiting CMF than the atmospheric pressure group. Among them, the Protease N of the HHP group had the highest capacity to inhibit CMF, with an inhibitory percentage of 30.1%, which was approximately 1.5 times that of the atmospheric pressure group. This result was similar to one in the study of pinto bean protein concentrate by Garcia-Mora et al. [27], in which pinto bean protein concentrate was hydrolyzed by Savinase under HHP to increase its antioxidant activity. The result may have been due to the fact that pinto protein is easily induced to expose more active cleavage sites under HHP, and the protein structure after HHP was also conducive to protease hydrolysis and the release of active peptides [27]. The results indicate that although the Protease N peptide content of the HHP group (597.4 mg/g) was lower than that of the Protease M and Peptidase R groups, Protease N under HHP can cause the sea bass protein to release peptides that have a stronger inhibitory capacity against CMF. Therefore, Protease N at 100 MPa was set as the condition for subsequent experiments to perform hydrolysis of sea bass byproducts and sample preparation.

### 2.2. Effects of HHP Time and Protease N Addition on Chemical Composition of Fermented Hydrolysates and CMF-Inhibitory Capacity

Our previous studies have uncovered that lactic acid bacteria can not only modify the undesirable bitter peptides in protein hydrolysates but also increase the content of functional peptides [17,18,19]. Therefore, in this study, we fermented sea bass byproducts with mixed lactic acid bacteria for 5 h. After the number of lactic acid bacteria reached 2.3 × 10^7^ CFU/mL and the pH of the fermented product was six (the pH condition for the optimal activity of Protease N is six to seven), Protease N was added to perform a 0, 3 (F-HHP-PN3), and 6 h (F-HHP-PN6) hydrolysis under HHP (100 MPa), and the hydrolysate composition and CMF-inhibitory capacity were analyzed and evaluated. The hydrolysates were analyzed through preference tasting evaluation. The findings indicated that the sea bass byproducts fermented by lactic acid bacteria and hydrolyzed under HHP did not have a bitter taste (data not shown). As Table 2 suggests, the soluble protein content increased after the sea bass byproducts were fermented by lactic acid bacteria and without protease (F-HHP) addition under HHP for 0 h, 3 h, and 6 h, which was 1.8 times of the 0 h sample (control group). The possible reason for the increase in soluble protein content was that the proteases secreted during the fermentation process of lactic acid bacteria can break insoluble proteins down into soluble proteins under high pressure, thus, the soluble protein content increased with HHP hydrolysis duration [36,37]. PepX peptidase purified from *S. thermophilus* and *L. bulgaricus* lysates was pressurized under HHP of 100 MPa at 20, 30, and 40 °C, and the peptidase still presented satisfactory enzyme activity [36,37,38].

The results for F-HHP-PN3 and F-HHP-PN6 indicate that the soluble protein, peptide, and free amino acid content levels were higher than those of the F-HHP group (Table 2). The content of peptides and that of free amino acids of F-HHP-PN0-6 increased with hydrolysis duration. After 6 h of hydrolysis, their levels were 421.9 mg/mL and 76.0 mg/mL, respectively, which were higher than that of the F-HHP-PN3 group by approximately 1.12 and 1.25 times. This indicates that the processing of fermented sea bass byproducts by using HHP-assisted protease hydrolysis was beneficial to increasing soluble protein content, and the byproducts can be further hydrolyzed into small molecules of peptides and free amino acids. Moreover, this study further evaluated the CMF-inhibitory capacity of F-HHP and F-HHP-PN at different hydrolysis durations (up to 6 h). The inhibitory percentage of the F-HHP0-6 group was 75.0–77.5%, and the inhibitory percentage of F-HHP-PN0-6 increased with the hydrolysis duration (75.0–88.4%). F-HHP-PN6 had the highest inhibitory percentage, 88.4%, which was equivalent to 1.2 times that of F-HHP0 and 1.13 times F-HHP-PN3. When cholestyramine was simultaneously used as the positive control group, its CMF-inhibitory percentage was 98.0 ± 0.6% (Table 2). Marques et al. [39] noted that the bioactive peptides produced in the hydrolysis of plant and animal proteins can reduce the absorption of exogenous cholesterol by destroying micelle formation in the intestine. Protein hydrolysates derived from animal sources including freshwater clam (*Corbicula fluminea*) (10 mg/mL), sericin (10 mg/mL), and milk protein (10 mg/mL) had CMF inhibitory percentages of 26% [15], 58% [3], and 37% [6], respectively. Peptides can inhibit CMF because their amino acid sequence is rich in hydrophobic amino acids, which can competitively bind with bile acid and can arrange cholesterol into a new structure, thereby destroying CMF and reducing the absorption of exogenous cholesterol content [40,41]. In addition, the effect of HHP on protein was reported mainly to make the noncovalent interactions (hydrophobic bond, ionic bond, and hydrogen bond) of the protein unstable. Among them, the hydrophobic interaction with a stable tertiary structure of the protein is the most sensitive to pressure. Therefore, the changing of protein structures and denaturation easily occur under HHP, which in turn exposes new cleavage sites to enhance the hydrolysis of proteins and to release active peptides with strong CMF-inhibitory capacities [34].

### 2.3. Effects of F-HHP-PN6–Simulated Gastrointestinal Digestive Protease Hydrolysis on CMF Inhibition

Biologically active peptides may change their activity after being digested by the gastrointestinal tract. Therefore, this study analyzed F-HHP-PN6 hydrolysis by simulating gastrointestinal digestive enzymes in vitro and evaluated its CMF-inhibitory capacity (Table 3). The findings signify that the CMF-inhibitory percentage of F-HHP-PN6 after being hydrolyzed by pepsin and pancreatin decreased from 88.4% to 47.7% and 42.1%, respectively. This result was similar to that for the protein isolate of olive seeds; after the fraction F3 with a molecular weight of >5000 Da was hydrolyzed by simulated gastrointestinal enzymes, the inhibitory percentage against CMF decreased from 29% to 10% [42]. Lin et al. [15] reported similar results. After freshwater clam hydrolysate was hydrolyzed by pepsin, its CMF-inhibitory percentage was also reduced, from 26.4% to 18.5%. The possible reason was that the biologically active peptides were hydrolyzed into less active peptides and amino acids after digestion by the stomach and the intestines, which decreased the inhibitory effect. Although the inhibitory percentage of the hydrolysate exhibited a downward trend after the gastrointestinal tract simulation, it still retained a CMF-inhibitory capacity. This result could help with understanding the active peptide sequence released by F-HHP-PN6 through gastrointestinal enzyme hydrolysis after oral administration and its stability assessment. Therefore, the hydrolysate (F-HHP-PN6-PP) of F-HHP-PN6 after being hydrolyzed by pepsin and pancreatin in sequence was used for peptide purification and sequence identification analysis.

### 2.4. Size Exclusion Chromatography of F-HHP-PN6-PP

**Figure 1:**
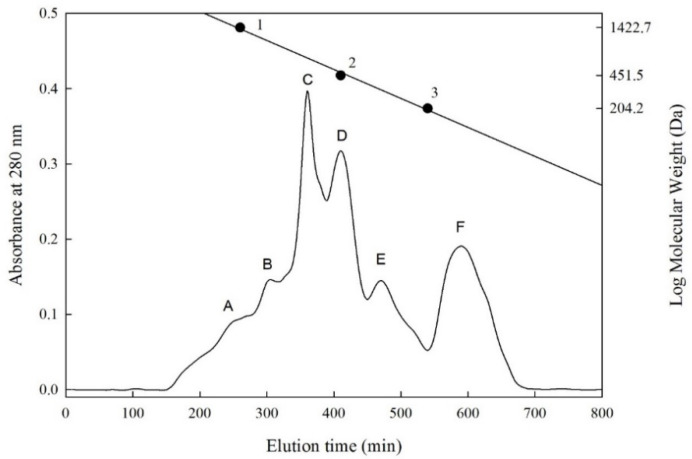
Sephadex G-25 column chromatography of peptides separated from F-HHP + PN-6 + PP. ● (Standard materials) 1, Bacitracin (1422.7 Da); 2, Glycine-Glycine-Tyrosine-Arginine (451.5 Da); 3, Tryptophan (204.2 Da). F-HHP + PN-6 + PP: lactic fermented seabass byproduct hydrolysates produced by Protease N under HHP for 6 h and then rehydrolysis with gastrointestinal proteases. (A–F: numbers of fractions).

We used Sephadex G-25 for size exclusion chromatography and purification analysis of the F-HHP-PN6-PP. According to the results of the size exclusion chromatography, six fractions A to F (Figure 1) were separated, with molecular weight ranges of 1280–1110, 1040–900, 730–640, 520–450, 340–290, and 140–130 Da. The peptide concentrations were 4.78, 8.52, 2.83, 0.75, 0.18, and 0.28 mg/mL, respectively. Fractions D, E, and F had higher CMF inhibitory percentages (50.3%, 43.0%, and 49.1%); fraction D had the highest (Table 4). The inhibitory percentage of each fraction and the peptide concentration were converted into inhibitory efficiency ratios (IER = inhibition [%]/peptide concentration [mg/mL]). Higher values represented stronger inhibitory capability. Among all the fractions, fraction E had the highest IER, 238.9%/mg/mL, and its molecular weight ranged from 340 to 290 Da, indicating that it was a di-, tri-, or tetrapeptide.

Shi et al. [43] measured the molecular weights of peptides in the size exclusion chromatography of chickpea protein hydrolysate. The CMF-inhibitory percentage at the weight of 5–6 kDa was 30.24%, whereas it was 47.83%, 63.44%, and 62.16%, respectively, at 3–5 kDa, 1–3 kDa, and <1 kDa. Shi et al. [43] reported a similar result, in which smaller peptide molecular weights (<1 to 3 kDa) tended to have higher CMF-inhibitory capacities. Our previous study revealed that among the size exclusion chromatography fractions (A to F; MW 340–2160 Da) of the freshwater clam hydrolysate, the molecular weight of fraction F was between 390 and 340 Da, and it had the highest CMF inhibitory percentage (33.3%), and the IER value was 831.5 %/mg/mL [15].

### 2.5. Separation and Purification of Peptides in Fraction E That Inhibit CMF

**Figure 2:**
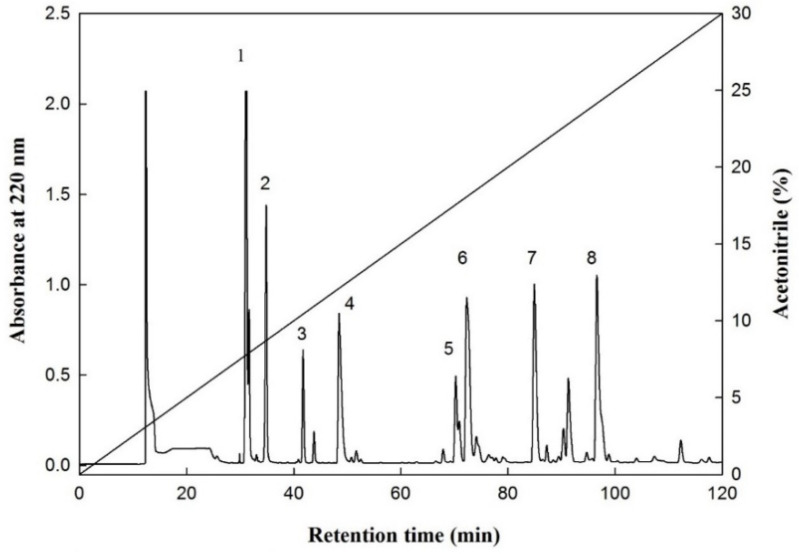
An elution profile of the fraction E from F-HHP + PN-6 + PP by reversed-phase HPLC. Column: Synergi 4u Hydro-RP 80A (250 × 10 mm; particle size, 4 μm; Phenomenex, Torrance, CA, USA); elution A (deionized water containing 0.1% trifluoroacetic acid (TFA) and B (100% acetonitrile containing 0.1% TFA); mobile phase: linear gradient from 0% to 30% of B within 120 min; and flow rate of 1.0 mL/min at room temperature, and detection at 220 nm. (1–8: numbers of the peaks).

The F-HHP-PN6-PP was processed through size exclusion chromatography to separate the Fraction E with the highest IER, and the peptide peak was further separated and purified using RP-HPLC. The chromatographic conditions were to use a semi-preparative C_18_ column, and the mobile phases were eluents A and B. Eluent A was deionized water containing 0.1% trifluoroacetic acid, and eluent B contained 0.1% trifluoroacetic acid in acetonitrile solution; both were eluted with a linear gradient of eluent A to 30% eluent B within 120 min, and the peaks were purified and collected. With different concentrations of acetonitrile, eight peaks of E_1_ to E_8_ at 7.9%, 8.7%, 10.3%, 11.9%, 17.2%, 17.5%, 20.9%, and 23.9% were eluted, respectively (Figure 2). The peaks eluted with higher concentrations of acetonitrile had stronger hydrophobicity. Kwon et al. [16] compared the CMF-inhibitory capacities of two synthetic peptides, Leu-Pro-Tyr-Pro-Arg (LPYPR) and Ser-Pro-Tyr-Pro-Arg (SPYPR), and determined that the effect of SPYPR was poorer. This may be due to the lower hydrophobicity of the peptide; its hydrophobicity was correlated to hypocholesterolemic activity. In the current study, the peptide concentration of each peak was analyzed simultaneously, and the results indicated that the peptide content of peaks E_1_, E_2_, and E_3_ was below the detection limit (0.01 mg/mL), thus, it may not be a peptide substance. Therefore, only E_4_ to E_8_ peaks were further analyzed and identified the amino acid sequence of the peptides.

### 2.6. Identification of Peptide Sequence

**Figure A1:**
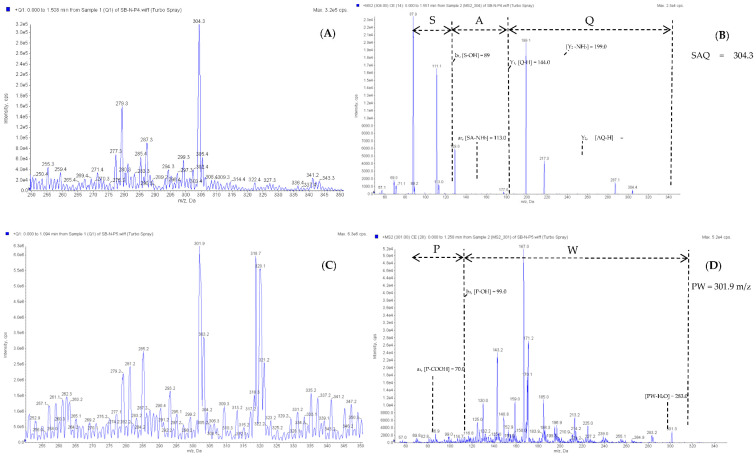
Identification of molecular mass and amino acid sequence of the peak E4 and E5 from F-HHP + PN6-PP by ESI/MS/MS. (**A**,**B**) the MS/MS spectra of peptide SAQ (*m*/*z* 304.3). (**C**,**D**) the MS/MS spectra of peptide PW (*m*/*z* 301.9).

**Figure A2:**
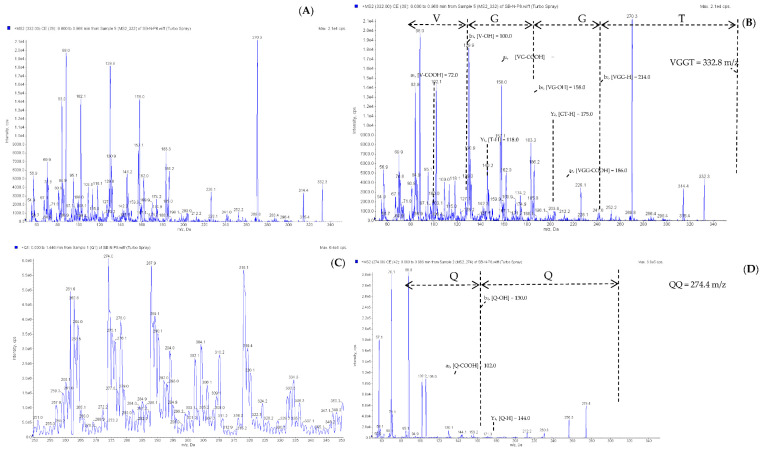
Identification of molecular mass and amino acid sequence of the peak E_6_ and E_8_ from F-HHP + PN6-PP by ESI/MS/MS. (**A**,**B**) the MS/MS spectra of peptide VGGT (*m*/*z* 332.8). (**C**,**D**) the MS/MS spectra of peptide QQ (*m*/*z* 274.0).

The E_4_ to E_8_ peaks were measured using the ESI tandem mass spectrometer to identify the peptide sequence, and the mass spectrometer scanned at a mass range of 250–350 *m*/*z* to obtain a mass spectrum. Subsequently, the mass-to-charge ratio signal was scanned and detected by the mass analyzer to obtain the secondary mass spectrum (Figure A1 and Figure A2 in Appendix A). The analysis performed using Analyst version 1.5.1 (AB Sciex) software identified the peptide sequence of E_4_ to E_8_, but no corresponding peptide sequence was identified for the E_7_ peak. The peak molecular weights of E_4_, E_5_, E_6_, and E_8_ were 304.3, 301.9, 332.8, and 274.4 *m*/*z*, respectively, and the peptide sequences were Ser-Ala-Gln (SAQ), Pro-Trp (PW), Val-Gly-Gly-Thr (VGGT), and Gln-Gln (QQ) (Table 5). These sequences, SAQ, PW, VGGT, and QQ, are the first to be reported for peptides that inhibit CMF activity. A subsequent search of the UniProt database revealed that the peptide sequences SAQ, PW, and QQ were mainly derived from lipoxygenase-5 (f 318-320), 5-HT receptor 1A (f 124-125), and chymotrypsinogen (f 36-37), but VGGT was not derived from sea bass protein (Table 5) [44]. Hence, we speculated that the VGGT sequence that was not aligned to the protein source may have derived from a peptide produced during the fermentation process of lactic acid bacteria.

To further understand the inhibitory capacity of SAQ, PW, VGGT, and QQ against CMF, the four peptide sequences were synthesized through solid-phase synthesis, and the concentration of each peptide was measured to estimate the IER (Table 5). However, QQ could not be synthesized (technically), and its activity could not be further measured. The results indicated that the IER values of SAQ, PW, and VGGT were 361.7, 3230.0, and 302.9%/mg/mL, respectively. According to the results, the inhibitory percentages of the three synthetic peptides against CMF were the highest in Pro-Trp. This may be because the Pro-Trp sequence contained amino acids with more hydrophobic groups, and the hydrophobic groups were successful in competing with bile acids and thus, in inhibiting CMF. Iwami et al. [45] demonstrated that peptides with high bile acid-binding capacities can inhibit the absorption of bile acids in the ileum, thereby reducing cholesterol concentration in the blood. These results support the hypothesis of Kwon et al. [16] who demonstrated and compared the CMF-inhibitory capacity of LPYP, and its two derivatives, LPYPR and SPYPR. SPYPR replaced the N-terminal amino acid in the hydrophobic leucine with the more hydrophilic serine. The results of feeding the three synthetic peptides to hypercholesterolemic mice signified a reduction in the total cholesterol concentration in the blood by 30%, 31%, and 14%, respectively. However, SPYPR had a weaker hypocholesterolemic effect, which may have been related to the reduced hydrophobicity of the peptide [16]. This finding was similar to the results for freshwater clam hydrolysates in our previous study [15]. The main peptide sequences in the freshwater clam hydrolysate inhibiting CMF were Val-Lys-Pro (VKP) and Val-Lys-Lys (VKK), with inhibitory capacities of 32.4% and 5.1%, respectively. Moreover, Zhong et al. [10] analyzed the soy protein hydrolysate through RP-HPLC chromatography and performed an online gradient (25–75% ethanol solution) for elution and analysis. The results indicated that 75% ethanol fraction contained more hydrophobic amino acids (i.e., Ile, Tyr, Phe, Pro, Leu, Val, and Lys) and had the strongest inhibitory effect on CMF. In addition, Megías et al. [41] hydrolyzed sunflower protein isolate by the proteases of Alcalase, pepsin, and pepsin + pancreatin. The active peptides in the hydrolysates that inhibited CMF all contained high levels of hydrophobic amino acid content (i.e., Ala, Tyr, Valine, and Leu). Other researchers have also identified the CMF inhibitory peptides containing Pro [16], Ala [6], or both Ala and Pro [10]. In the present study, the amino acids in the hypocholesterolemic peptides (i.e., Ser-Ala-Gln, Pro-Trp, and Val-Gly-Gly-Thr) included Aal, Pro, and highly hydrophobic amino acids such as Val. However, the related mechanism of peptides in hypocholesterolemic activity has not yet been fully clarified. Future studies must consider the chemical properties and hydrophobic–hydrophilic balance of peptides, as well as the amino acid structure and molecular weight of the peptides, to further understand their effect on hypocholesterolemic activity.

## 3. Materials and Methods

### 3.1. Materials

The byproducts of sea bass (e.g., fish heads, fish bones, and tails after gill removal) were obtained from Liang Shing Frozen Seafoods Co., Ltd. (Pingtung County, Taiwan) and stored at −20 °C freezer for subsequent use. Protease N, Protease M, and Peptidase R with a nominal activity level of 150,000, 40,000, and 420 U/g, respectively, were supplied by Amano Enzyme Inc. (Yokohama, Nagoya, Japan). Freeze-dried mixed powders of lactic acid bacteria (*Lactobacillus casei*, *Lactobacillus acidophilus*, *Lactococcus latis subspecies lactise*, *Lactococcus latis* subspecies *cremoris*, *Lactococcus latis subsp lavtis bv. diacetylactis*, *Saccharomyces cerevisiae*, and *Saccharomyces lactis*) were purchased from Lyo-San Inc. (Lachute, QC, Canada). Digestive enzymes (pepsin and pancreatin) and other chemicals of analytical grade were obtained from Sigma Chemical Co. (St. Louis, MO, USA).

### 3.2. Preparation of Sea Bass Byproducts through HHP to Facilitate Protease Hydrolysis

Deionized water was added to 1 kg of sea bass byproducts (solid-to-liquid ratio of 1:2). The byproducts were homogenized for 2 min in a high-speed mixer (Vita-mix TNC5200, Vita-Mix Co., Cleveland, OH, USA) to produce a sea bass paste. The paste was packed and sealed in a vacuum bag (Nylon/PE) and then sterilized in a constant temperature water bath at 90 °C for 10 min. After sterilization, the enzyme was added to the paste (substrate) at ratios of 1:100 (*w*/*w*). After preparation with deionized water to a concentration of 1%, the commercial proteases (Protease N, Protease M, and Peptidase R) were filtered and sterilized with a 0.2 μm filter membrane under HHP (TFS-100, Toyo-Koatsu Innoway Co. Ltd., Nishiku, Hiroshima, Japan) of 100 MPa and atmospheric pressure of 0.1 MPa at 50 °C for 24 h for hydrolysis. The hydrolysates were inactivated at 90 °C for 10 min after the reaction was completed. After centrifugation at 2710× *g* (CR21, Hitachi Co. Ltd., Minato-ku, Tokyo, Japan) for 30 min, the filtrate filtered with No. 2 filter paper was then freeze-dried into powder for experimental use.

### 3.3. Preparation of Fermented Sea Bass Byproducts through HHP to Facilitate Protease Hydrolysis

Sterilized sea bass paste was prepared according to the aforementioned method. The sea bass paste was mixed with 0.1% lactic acid bacteria powder (*w*/*v*, based on the total volume of fermentation broth) and fermented at 42 °C for 5 h to make the bacteria count reach 2.3 × 10^7^ colony-forming unit/mL. The fermented sea bass paste was added with 1% Protease N (*w*/*w*, based on the solid content of the paste as the substrate, prepared with deionized water to a concentration of 1% and then filtered and sterilized with a 0.2 μm filter membrane) for reaction at 50 °C for 0, 3, and 6 h under HHP of 100 MPa (HHP-assisted Protease N hydrolysis after fermentation, F-HHP-PN0 through -6). Sterilized sea bass paste fermented by lactic acid bacteria without adding protease was used as the control group: F-HHP (HHP treatment without protease after fermentation). Finally, the protease reaction was terminated by heating at 90 °C for 10 min, and then the paste was centrifuged at 2710× *g* for 30 min (CR21, Hitachi). The filtrate was filtered with No. 2 filter paper and freeze-dried into a powder for subsequent experimental use.

### 3.4. Determination of Free Amino Acid Content 

The analysis of free amino acids was conducted in accordance with described methods, with slight modifications [46,47]. First, 0.5 mL of the diluted solution of sea bass protein hydrolysate (50 mg/mL) was added with 1.0 mL of Cd-ninhydrin reagent to a water bath at 84 °C for 5 min. The solution was cooled to room temperature quickly in the water bath, and the absorbance value was measured at 507 nm with a spectrophotometer (EVOLUTION 60S, Thermo Fisher Scientific Inc., Waltham, MA, USA). Finally, the obtained leucine standard calibration curve was converted to free amino acid content.

### 3.5. Determination of Soluble Protein Content 

The measurement of soluble protein content was slightly modified from the Folin–Lowry method [47,48,49]. First, 0.1 mL of solution was extracted from the sea bass protein hydrolysate (50 mg/mL), and 0.5 mL of reagent A and 4 mL of reagent B (Bio-Rad Dc Protein Assay Kit, Bio-Rad Labortories Inc., Berkeley, CA, USA) were added to it. The solution was shaken and mixed well and let stand at room temperature for 15 min. Finally, its absorbance value was measured at 540 nm (EVOLUTION 60S, Thermo Fisher Scientific). The standard calibration curve was obtained with bovine serum albumin as the standard for analysis and was converted into the soluble protein content in the sample.

### 3.6. Determination of Peptide

For the analysis of peptide content, the determination method of Church et al. [50] and Lin et al. [47] was used, with slight modifications. *O*-phthaldialdehyde reagent and a standard calibration curve obtained from Leu-Gly reference material were used to convert the peptide content. Before the measurement, the sample solution (50 mg/mL) was taken and filtered with a 0.22 μm membrane, and 50 μL of the filtrate was added to the mixed solution containing 2 mL of *o*-phthaldialdehyde. After shaking and mixing, it was let stand at room temperature for 2 min, and then a spectrophotometer was used to measure the absorbance value at 340 nm (EVOLUTION 60S, Thermo Fisher Scientific).

### 3.7. Inhibition of CMF

Measurement of the inhibitory capacity of sea bass protein hydrolysate and cholestyramine on CMF was according to the methods of Nagaoka et al. [6] and Lin et al. [15] with slight modifications. A 70 mg of sample was dissolved in 7 mL of micellar solution (formulation mixture containing 10 mM sodium taurocholate (Sigma Chemical., Co.), 2 mM cholesterol (Sigma Chemical., Co.), 5 mM oleic acid, 132 mM sodium chloride, and 15 mM sodium phosphate (pH 7.4)), and the homogeneous mixture was prepared with ultrasound (Sonicator S3000, Misonix Co. Ltd., Farmingdale, NY, USA, power, 220 W) for 2 min. The mixture was reacted at 37 °C for 24 h and then centrifuged in an ultrahigh speed centrifuge (Hitachi SCP 85G, P65A Rotor) at 400,000 rpm and 20 °C for 60 min. Subsequently, 10 μL of the supernatant was collected, the absorbance was measured at 500 nm with cholesterol enzymatic kits (Auid Diagnostics, Cork, Ireland), and the cholesterol content in the micelles was converted. Each sample was analyzed in triplicate. Finally, the inhibition of CMF of the supernatant fraction is calculated in accordance with the equation [(BC − RS)/ BC] × 100, where RS is the cholesterol content of the reaction mixture (sample, s), in reference to the buffer (control, c).

### 3.8. In Vitro Gastrointestinal Digestion 

Digestion was simulated in vitro with slight modifications of previously published methods [15,51]. The sample powder of F-HHP-PN6 (45 g) was taken and dissolved in 1500 mL of 0.1 M KCl-HCl (pH 2.0) buffer solution (*w*/*v*). Pepsin (EC 3.423.1; 1:10,000) was added to make the ratio of protease to protein (E/S = 1/25, [*w*/*w*]) and let react at 37 °C for 4 h. The pH was then adjusted to 7.0 with 2N sodium hydroxide to stop the reaction. The portion of hydrolysate (250 mL) was removed and heated in a boiling water bath for 10 min to deactivate the protease. Subsequently, pancreatin (E/S = 1/25, [*w*/*w*]) was added to the remaining hydrolysate for reaction at 37 °C for 4 h, and then the mixture was heated in a boiling water bath for 10 min to terminate the reaction. The hydrolysate was centrifuged at 6930× *g* (Hitachi high refrigerated centrifuge CR21) for 30 min, and the supernatant was freeze-dried into a powder for subsequent use (F-HHP-PN6-PP).

### 3.9. Size Exclusion Chromatography Analysis of Sea Bass Protein Hydrolysate 

The size exclusion chromatography of sea bass protein hydrolysate was performed on the basis of the method of Chen et al. [17] and Lin et al. [15] with minor modifications. The freeze-dried hydrolysate F-HHP-PN-6-PP with the highest CMF inhibitory activity was purified and separated by using size exclusion chromatography (Sephadex G-25 column, 1.6 × 90 cm; Amersham Pharmacia Biotech AB, Uppsala, Sweden) and equilibrated with 0.02% sodium azide deionized water. The F-HHP-PN6-PP sample powder was prepared at a concentration of 30 mg/mL. After filtration by a 5000 Da filter membrane, 2 mL of the filtrate was used as the injection volume; the 0.02% sodium azide aqueous solution was used as the eluent, and the constant flow rate of the peristaltic pump (Gilson MP1/LF, Gillson Medical Electronics, Villiers-le-Bel, France) was 0.5 mL/min. A liquid collector (FC203B, Gillson Medical Electronics) was used to collect the eluate (5 mL/tube), and absorbance of the eluate was measured at 280 nm. Finally, the elution time was plotted as the horizontal axis and the absorbance value as the vertical axis, and the molecular weight of the sample obtained from the standard calibration curve was converted. The molecular weights of the standards were as follows: bacitracin: 1422.0 Da; glycine-glycine-tyrosine-arginine: 451.5 Da; and tryptophan: 204.2 Da.

### 3.10. Purification of the CMF-Inhibitory Peptide

Purification of the CMF-inhibitory peptide from protein hydrolysate was performed by following the method described by Lin et al. [15] and Lin et al. [52] with minor modifications. The fraction with the highest CMF inhibitory activity in the size exclusion chromatography (Sephadex-25) was collected and freeze-dried, and the peptide was purified and analyzed through RP-HPLC (model L-7100, Hitachi) using a semi-preparative C_18_ column. The column chromatography was a reversed-phase C_18_ column (Synergi 4 μ Hydro-RP 80 Å, 10 × 250 mm^2^; particle size: 4 μm; Phenomenex, Torrance, CA, USA). The chromatographic conditions were as follows: eluent A: 0.1% trifluoroacetic acid in deionized water; eluent B: 0.1% trifluoroacetic acid in acetonitrile solution as the mobile phase. The concentration of eluent B was increased from 0% to 30% within 120 min for linear gradient elution, at a flow rate of 1.0 mL/min. The sample injection volume was 500 μL, with a wavelength of 220 nm (GILSON151/VIS Detectors, Gillson Medical Electronics were used for testing and connected to a data station, 715 system controller, Gilson Medical Electronics). The main peaks with high CMF inhibitory activity were collected, and each peak at a flow rate of 1.5 mL/min was set. The sample injection volume was 10 μL and the column C_12_ with lower hydrophobicity was conducted under the mentioned gradient conditions (Joupiter 4 µm Proteo 90 A, 250 × 4.6 mm^2^, Phenomenex). After column chromatography analysis confirmed that the collected material was a single peak, the confirmed peaks were collected and freeze-dried for peptide sequence analysis.

### 3.11. ESI/MS/MS and Peptide Data Analyses

The peptide sequence analysis used the RP-HPLC peptide purification, collection, and ESI/MS/MS peptide sequence identification methods described by Lin et al. [15,52] and Huang et al. [53] with minor modifications. Before purifying, identifying, and analyzing of the peptide sequence, the concentration of the prepared sample had to first be increased. The concentration of F-HHP-PN6-PP was increased from 30 to 75 mg/mL, and the fraction E with the highest inhibitory percentage was purified and collected through size exclusion chromatography on a Sephadex G-25 column. The fraction E collected by two separations of chromatography and the combined collection were freeze-dried into a powder and dissolved in 0.5 mL of deionized water. Subsequently, the aforementioned RP-HPLC method was used to further purify and separate the peaks of the peptides on a semi-preparative C_18_ column. Later, the main peaks from RP-HPLC chromatography were repeatedly collected (five times), and then the analytical C_12_ column was used to confirm that each peak was a single component through the aforementioned method. The mixtures collected five times were then freeze-dried into powders and their inhibitory capacities in CMF confirmed. In addition, the peptide sequence was analyzed through ESI-MS/MS (AB Sciex Instruments QTRAP 5500, AB Sciex Pte. Ltd., Concord, ON, Canada). Finally, the freeze-dried powder of the abovementioned peaks was prepared with deionized water into a sample solution with a concentration of 1000 pmol/μL. The sample (100 μL) was injected into the tandem mass spectrometer. The ion source used electrospray ionization (ESI) to ionize, and then a quadrupole ion trap was used as a mass analyzer to select the precursor ions and record the *m*/*z* value of the precursor ions in the positive charged mode (5500 V; MS range: 250–350 *m*/*z*) to obtain a mass spectrum. Subsequently, the precursor ion with the highest signal peak in the mass spectrum was selected and introduced into the collision chamber, and helium at a flow rate of 7 μL/min was used as the collision gas. Collision-induced dissociation was used to induce molecular fragmentation to generate product ion fragments, and then the *m*/*z* value and the number of product ions were analyzed through tandem mass spectrometry (MS/MS range: 50–350 *m*/*z*) to obtain the secondary mass spectrum. The mass spectrum was analyzed and compared to obtain the amino acid sequence of peptides by using Analyst version 1.5.1 (AB Sciex). The identified peptide sequences were aligned using the UniProt database to verify the amino acid sequences of the sea bass protein [44], and pairwise sequence alignment tools were used to confirm the sequences [54]. After alignment, the identified peptide sequences were lipoxygenase-5 (accession number: A0A160E7B4), 5-HT receptor 1A (accession number: A8D3L1), and chymotrypsinogen (accession number: K9MXV0). Finally, the identified active peptides were synthesized through solid-phase peptide synthesis, and their CMF-inhibitory activity was measured. In addition, an RP-HPLC column (ODS C_12_) was used for the qualitative analysis of these peptides in sea bass hydrolysate by using the described method, with the synthetic peptides as standards.

### 3.12. Statistical Analysis of Data

Except for the analytical data of protein hydrolysate yield, size exclusion chromatography, and RP-HPLC chromatography that were presented as the mean of triplicates, the experimental data of each item were presented in mean ± SD. SAS software [55] was employed to analyze the differences in the aforementioned experimental results through a general linear model procedure. Moreover, Duncan’s multiple range test was used to compare the means of multiple groups.

## 4. Conclusions

The sea bass byproducts were fermented via lactic acid bacteria, and the hydrolysates prepared through protease N hydrolysis (F-HHP-PN6) under HHP of over 100 MPa for 6 h was beneficial for the release of the contents of soluble protein, peptides, and free amino acids. Moreover, their CMF inhibition rate reached 88.4%, which was approximately 1.2 times higher than that of the control group, F-HHP0. The F-HHP-PN6 hydrolysates passing through the simulated gastrointestinal digestion was further purified; the main peptide sequences inhibiting CMF were identified as Ser-Ala-Gln, Pro-Trp, and Val-Gly-Gly-Thr, with inhibition efficiency ratios 361.7, 3230.0, and 302.9 %/mg/mL, respectively. This study suggested that an CMF-inhibitor could be derived from a protein hydrolysate of sea bass byproducts and use to serve as a basis for developing functional food that regulate blood cholesterol.

## Figures and Tables

**Table 1:** Effect of proteases and HHP on chemical compositions and the inhibition against cholesterol micelle formation of seabass byproduct hydrolysates *.

Pressure (MPa)	Enzymes ^†^	Soluble Protein (mg/g)	Peptide Content (mg/g)	Free Amino Acid (mg/g)	Inhibition ^‡^ (%)
	**Cholestyramine** **(Positive Control)**	— ^§^	—	—	98.0 ± 0.6 ^a^
0.1	Protease N	654.4 ± 2.8 ^b^	656.2 ± 1.8 ^e^	157.0 ± 0.4 ^d^	15.9 ± 2.1 ^e^
	Protease M	409.9 ± 12.2 ^d^	865.0 ± 6.6 ^c^	308.7 ± 2.2 ^c^	16.4 ± 1.3 ^de^
	Peptidase R	439.6 ± 19.5 ^c^	737.0 ± 12.1 ^d^	306.4 ± 0.9 ^c^	17.0 ± 0.1^de^
100	Protease N	696.8 ± 11.0 ^a^	597.4 ± 3.1 ^f^	138.4 ± 0.6 ^e^	30.1 ± 0.1 ^b^
	Protease M	342.0 ± 11.0 ^e^	908.6 ± 6.7 ^a^	359.8 ± 1.9 ^a^	22.8 ± 0.6 ^c^
	Peptidase R	329.2 ± 4.5 ^e^	806.4 ± 8.8 ^b^	350.0 ± 3.1 ^b^	21.4 ± 4.8 ^cd^

* Means ± standard deviation (*n* = 3). Different superscripts (a–f) in the same column indicate significant difference (*p* < 0.05) between samples. ^†^ Enzymatic hydrolysis at 50 °C for 24 h. ^‡^ The inhibition (%) against cholesterol micelle formation was measured using the concentration 10 mg/mL of each sample powder. ^§^ Undetected.

**Table 2:** Effect of hydrolysis time during HHP on chemical compositions and the inhibition against cholesterol micelle formation from lactic fermentation seabass byproduct hydrolysates *.

Sample	Hydrolysis Time (h)	Soluble Protein (mg/g)	Peptide Content (mg/g)	Free Amino Acid (mg/g)	Inhibition (%)
	**Cholestyramine** **(Positive Control)**	— ^§^	—	—	98.0 ± 0.6 ^a^
F-HHP ^†^	0	187.1 ± 7.9 ^e^	112.8 ± 2.0 ^c^	30.1 ± 0.0 ^c^	75.0 ± 1.4 ^d^
	3	270.3 ± 1.8 ^d^	117.2 ± 4.0 ^c^	30.1 ± 0.0 ^c^	77.1 ± 0.5 ^cd^
	6	337.5 ± 0.8 ^c^	94.7 ± 1.6 ^d^	25.2 ± 0.3 ^d^	77.5 ± 1.1 ^c^
F-HHP + PN ^‡^	3	617.1 ± 1.6 ^a^	376.8 ± 4.9 ^b^	60.6 ± 0.0 ^b^	78.1 ± 0.2 ^c^
	6	565.8 ± 2.0 ^b^	421.9 ± 3.7 ^a^	76.0 ± 0.9 ^a^	88.4 ± 1.0 ^b^

* Means ± standard deviation (*n* = 3). Different superscripts (a–e) in the same column indicate significant difference (*p* < 0.05) between samples. ^†^ F-HHP: lactic fermented seabass byproduct hydrolysates produced by without adding Protease N under HHP for 0, 3 and 6 h. ^‡^ F-HHP + PN: lactic fermented seabass byproduct hydrolysates produced by Protease N under HHP for 3 and 6 h. ^§^ Undetected.

**Table 3:** Effect of digestion by gastrointestinal proteases on the inhibition against cholesterol micelle formation of F-HHP + PN-6 *.

Sample	Before Gastrointestinal Proteas Digestion of Inhibition (%)	After Gastrointestinal Proteases Digestion of Inhibition (%)	
		Pepsin	Pepsin + Pancreatin
Choletstyramine (positive control)	98.0 ± 0.6	— ^‡^	—
F-HHP + PN-6 ^†^	88.4 ± 1.0 ^a^	47.7 ± 0.7 ^b^	42.1 ± 2.1 ^c^

* Means ± standard deviation (*n* = 3). Different superscripts (a–c) in the same row indicate significant difference (*p* < 0.05) between samples. ^†^ F-HHP + PN-6: lactic fermented seabass byproduct hydrolysates produced by Protease N under HHP for 6 h. ^‡^ Undetected.

**Table 4:** The inhibition against cholesterol micelle formation of the size exclusion chromatographic fractions obtained from F-HHP + PN-6 + PP ^†^.

Fraction	Molecular Weight (Da)	Inhibition (%)	Peptide Concentration (mg/mL)	IER ^‡^ (%/mg/mL)
A	1280–1110	15.4	4.78	3.2
B	1040–900	28.1	8.52	3.3
C	730–640	39.1	2.83	13.8
D	520–450	50.3	0.75	67.1
E	340–290	43.0	0.18	238.9
F	140–130	49.1	0.28	175.4

^†^ F-HHP + PN-6 + PP: lactic fermented seabass byproduct hydrolysates produced by Protease N under HHP for 6 h and then rehydrolysis with gastrointestinal proteases. ^‡^ IER: Inhibitory efficiency ratio = Inhibition (%)/Peptide concentration (mg/mL).

**Table 5:** Amino acid sequence and inhibition against cholesterol micelle formation of peak E_4_, E_5_, E_6_, and E_8_ from fraction E of F-HHP + PN-6 + PP *.

Peak	Sequence	Inhibition (%)	Peptide Concentration ( mg/mL)	IER (%/mg/mL)	Origin ^†^
E_4_	Ser-Ala-Gln	21.7 ± 4.0	0.06 ± 0.0	361.7	Lipoxygenase-5, f 318-320
E_5_	Pro-Trp	32.3 ± 3.0	0.01 ± 0.1	3230.0	5-HT receptor 1A, f 124-125
E_6_	Val-Gly-Gly-Thr	21.2 ± 0.4	0.07 ± 0.0	302.9	—
E_7_	— ^‡^	—	—	—	—
E_8_	Gln-Gln	n.d. ^§^	n.d.	n.d.	Chymotrypsinogen, f 36-37

* F-HHP + PN-6 + PP: lactic fermented seabass byproduct hydrolysates produced by Protease N under HHP for 6 h and then rehydrolysis with gastrointestinal proteases. ^†^ The peptide sequence alignment of *Lates calcarifer* proteins (Lipoxygenase-5, 5-HT receptor 1A and Chymotrypsinogen). ^‡^ Non detected possible sequence. ^§^ Non detected.

## Data Availability

Not applicable.
